# The preparation and application of calcium phosphate biomedical composites in filling of weight-bearing bone defects

**DOI:** 10.1038/s41598-021-83941-3

**Published:** 2021-02-19

**Authors:** Lijia Cheng, Tianchang Lin, Ahmad Taha Khalaf, Yamei Zhang, Hongyan He, Liming Yang, Shuo Yan, Jiang Zhu, Zheng Shi

**Affiliations:** 1 College of Basic Medicine & Clinical Medical College & Affiliated Hospital of Chengdu University, Chengdu, 610106 China; 2 Department of Orthopedics, The First People’s Hospital of Chengdu, Chengdu, 610000 China

**Keywords:** Biomaterials, Experimental models of disease

## Abstract

Nowadays, artificial bone materials have been widely applied in the filling of non-weight bearing bone defects, but scarcely ever in weight-bearing bone defects. This study aims to develop an artificial bone with excellent mechanical properties and good osteogenic capability. Firstly, the collagen-thermosensitive hydrogel-calcium phosphate (CTC) composites were prepared as follows: dissolving thermosensitive hydrogel at 4 °C, then mixing with type I collagen as well as tricalcium phosphate (CaP) powder, and moulding the composites at 37 °C. Next, the CTC composites were subjected to evaluate for their chemical composition, micro morphology, pore size, Shore durometer, porosity and water absorption ability. Following this, the CTC composites were implanted into the muscle of mice while the 70% hydroxyapatite/30% β-tricalcium phosphate (HA/TCP) biomaterials were set as the control group; 8 weeks later, the osteoinductive abilities of biomaterials were detected by histological staining. Finally, the CTC and HA/TCP biomaterials were used to fill the large segments of tibia defects in mice. The bone repairing and load-bearing abilities of materials were evaluated by histological staining, X-ray and micro-CT at week 8. Both the CTC and HA/TCP biomaterials could induce ectopic bone formation in mice; however, the CTC composites tended to produce larger areas of bone and bone marrow tissues than HA/TCP. Simultaneously, bone-repairing experiments showed that HA/TCP biomaterials were easily crushed or pushed out by new bone growth as the material has a poor hardness. In comparison, the CTC composites could be replaced gradually by newly formed bone and repair larger segments of bone defects. The CTC composites trialled in this study have better mechanical properties, osteoinductivity and weight-bearing capacity than HA/TCP. The CTC composites provide an experimental foundation for the synthesis of artificial bone and a new option for orthopedic patients.

## Introduction

Currently, the global orthobiologics market could be segmented into the demineralized bone matrix (DBM), allograft, bone morphogenetic protein (BMP) and artificial bone graft substitutes, in which calcium phosphate (CaP) biomaterials were considered as an ideal artificial bone substitute, due to their compositional similarity with bone mineral^1–4^. Accumulating studies have shown that the CaP artificial bone that was widely applied in the filling of non-weight bearing bone defects was fragile; while the autografts remained the gold standard in the filling of weight-bearing bone defects. However, the autografts were very limited as, with the addition of immune rejection reactions, their application was restricted^5^. In order to enhance the hardness and improve the elastic modulus of the CaP biomaterials, some polymerides were added to synthesize composites, such as collagen, chitosan, hyaluronic acid, polylactic acid (PLA), polyglycolic acid (PGA), etc.^6–8^. For example, Danoux et al.^9^ prepared composites with PLA as a polymeric matrix and calcium carbonate or sodium phosphate salts as fillers. They found that it could promote human mesenchymal stem cell osteogenic differentiation and extracellular matrix mineralization. Makarov et al.^10^ found that CaP-polycaprolactone composite beads loaded with vancomycin could treat osteomyelitis. Moreover, Fang et al.^11^ developed a bio-composite bone cement composed of TCP, chitosan and acrylic, and such materials had better osteointegration than pure polymethyl methacrylate cement. However, there is still insufficient evidence around the usage of bio-composites in filling the load-bearing bone defects in clinical or preclinical studies.

**Figure 1:**
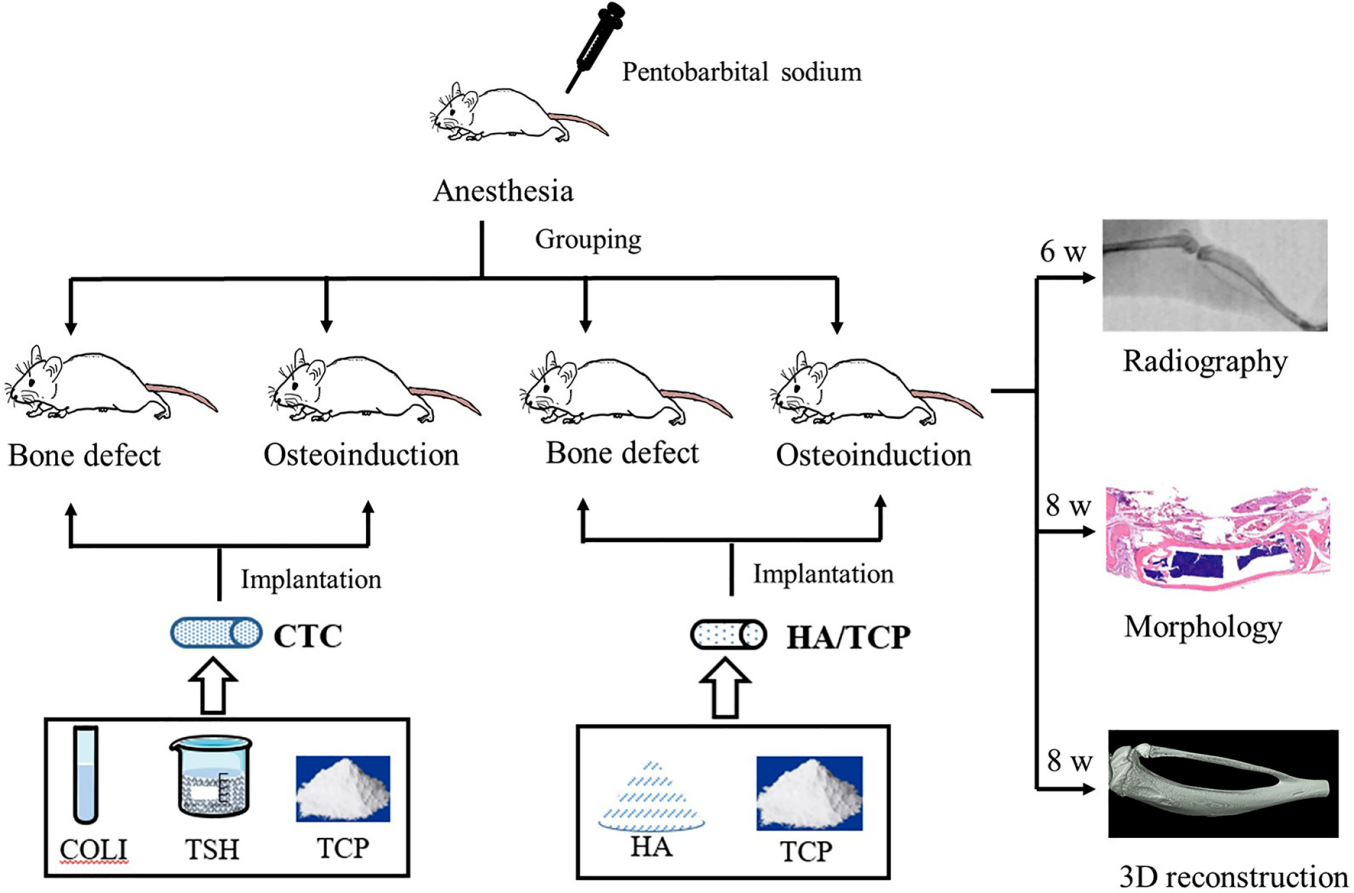
The overall idea of the experiment of this study.

This study takes advantage of thermosensitive hydrogel, type I collagen, and CaP powders as the raw materials to synthesize the CTC composites. The thermosensitive hydrogel was polylactic acid-polyethylene glycol-polylactic acid (PLGA-PEG-PLGA) polymer: it could dissolve in water to form a liquid when the temperature fell below the phase inversion temperature (PIT). Conversely, the liquid turned into a gel shape when the temperature rose above the PIT, and the process was reversible^12,13^. Collagen I was mainly a mineral secreted by osteoblasts, that could promote bone regeneration in bone tissue engineering^14,15^. As previously mentioned, CaP material was an excellent artificial bone: we speculated that the hardness and elasticity of the bio-composites would be improved with the help of thermosensitive hydrogel and collagen I. In a further study, the CTC composites would be implanted in muscle to induce ectopic bone formation and filled in large segments of tibia defects in mice. In order to verify the osteoinductivity, bone-repairing and load-bearing capacities of the newly synthesized composites. And the overall idea of the experiment is shown in the Fig. 1.

## Materials and methods

### Materials preparation

**Figure 2:**
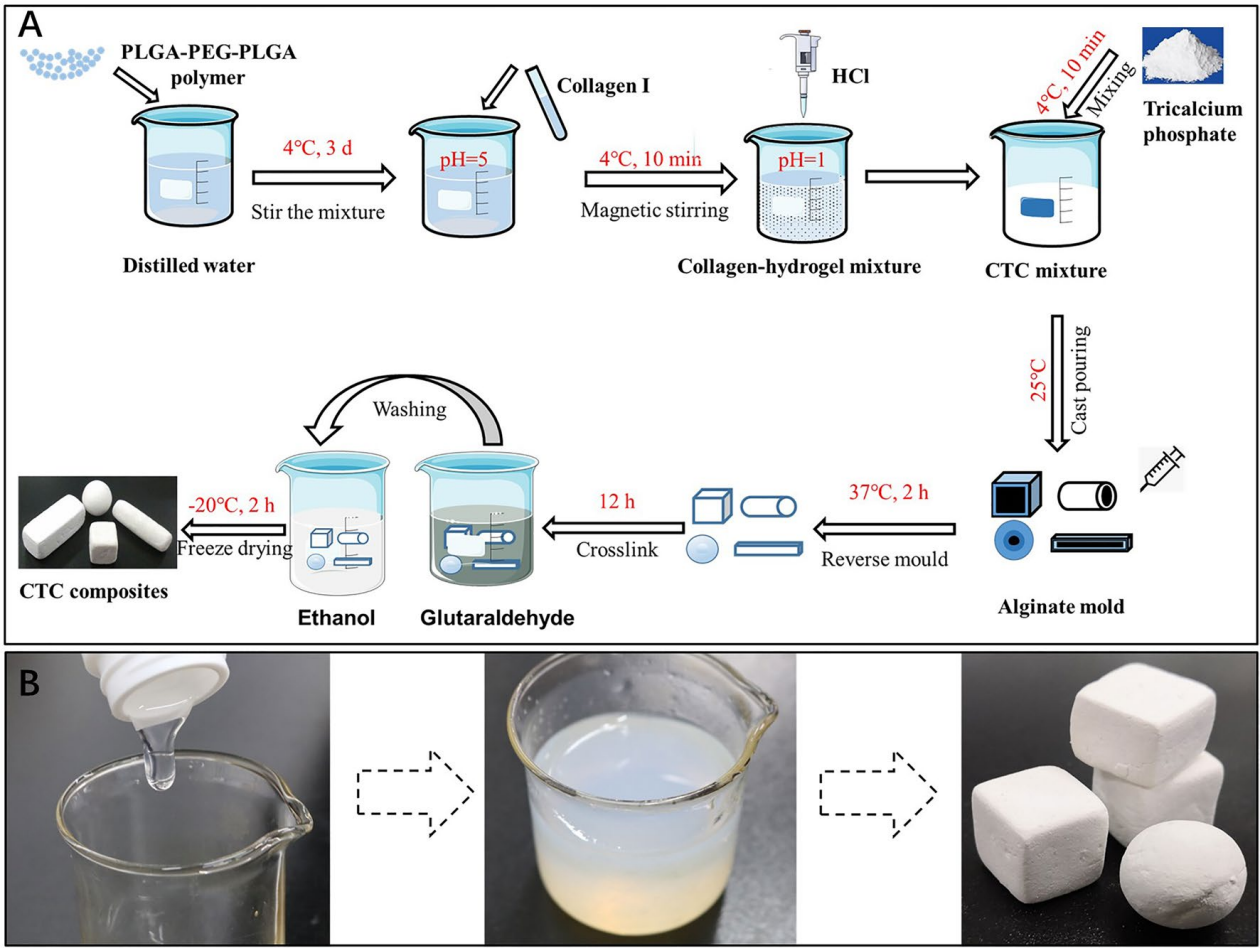
The schematic diagram and process of CTC bio-composite preparation. (**A**) The schematic diagram of material preparation. The ratio of collagen I, thermosensitive hydrogel and calcium phosphate was 25:15:60, and the materials could be molded into a mold. (**B**) The preparation process of CTC composites: the dissolution of the thermosensitive hydrogel, a mixture of three raw materials, solidification and shaping in a special mold.

Firstly, the thermosensitive hydrogel (PLGA-PEG-PLGA polymer, Daigang Biomaterial, China) was dissolved in double distilled water to form the hydrogel solution at 4 °C for 3 days; secondly, the type I collagen from rat tail (Sigma-Aldrich, USA) was added into hydrogel solution after dissolving in 5% acetic acid as a ratio of 15:25, then the dilute hydrochloric acid was added to adjust the pH 1 after stirring for 10 min; the mixture of the collagen-thermosensitive hydrogel was obtained. Thirdly, the tricalcium phosphate powder (Ca_3_(PO_4_)_2_, CaP, Sigma-Aldrich, USA) was weighed and mixed with the collagen-thermosensitive hydrogel solution for 10 min according to the proportion of 40:60, to get a collagen-thermosensitive hydrogel-calcium phosphate (CTC) mixture and the final ratio of the CTC composite was 25% collagen:15% thermosensitive hydrogel:60% calcium phosphate. The above procedures were performed at 4 °C to maintain the mixture hydrogel status. Lastly, this mixed slurry was poured into the mold and got solidification at 37 °C, then crosslinked with 2.5% glutaraldehyde for 12 h, washed with 95% alcohol, freeze-dried for 2 h at − 20 °C. Following the above steps, the CTC bio-composite was fabricated. The schematic diagram and process of material preparation are shown in Fig. 2. The conventional hydroxyapatite/β-tricalcium phosphate (HA/TCP) biomaterials contained 70 wt% HA and 30 wt% β-TCP were prepared as follows: the ceramic powders were prepared by wet method using Ca(NO_3_)_2_·4H_2_O and (NH_4_)_2_HPO_4_ as raw materials, then 10% H_2_O_2_ as foaming agent was mixed with powders to form slurry, which was heated for foaming at 70–80 °C. Finally, the mixed slurry was sintering at 1150 °C for 2 h to produce the porous HA/TCP biomaterials.

### Physicochemical characteristics of the CTC composites

#### X ray diffraction (XRD)

The CTC composites were grinded to a fine powder in a mortar and passed through a 320-mesh sieve, and the chemical composition of CTC, HA and TCP were evaluated with X-ray diffractometer (Analysis and Testing Center, Sichuan University, China).

#### Micro morphology and pore size

The specimens were cut in the middle, sprayed with gold–palladium-coated and the micro morphology and pore size were observed at different magnification under scanning electron microscope (SEM, National Engineering Research Center for Biomaterials, Sichuan University, China).

#### Shore durometer

The materials were prepared more than 2 cm $\times$ 2 cm $\times$ 0.5 cm and performed with Shore durometer D. The reference standard was GB/T 2411-2008.

#### Porosity

The porosity of the materials was measured by liquid displacement method. The materials were placed into a measuring cylinder with 20 ml anhydrous ethanol, the volume of ethanol was recorded as V1; 10 min after material immersion, measure the volume again (V2); next, the materials were taken out and the residual ethanol volume was measured (V3). The porosity (Po) was calculated as the following equation: Po% = (V1–V3)/(V2–V3) × 100%.

#### Water absorption ability

The water absorption ability reflected the hydrophilic and hydrophobic properties of materials. The fine particles of specimen surface were removed, then the materials were dried in oven at 100 °C for 2 h, cooled to the room temperature in a dryer, and weighed the mass of specimen (M1). Next, the samples were put into distilled water for 24 h, weighed the mass of specimen (M2) again. The water absorption (Wa) was calculated as the following equation: Wa% = [(M2 − M1)/M1] × 100%.

### Animal surgery

**Figure 3:**
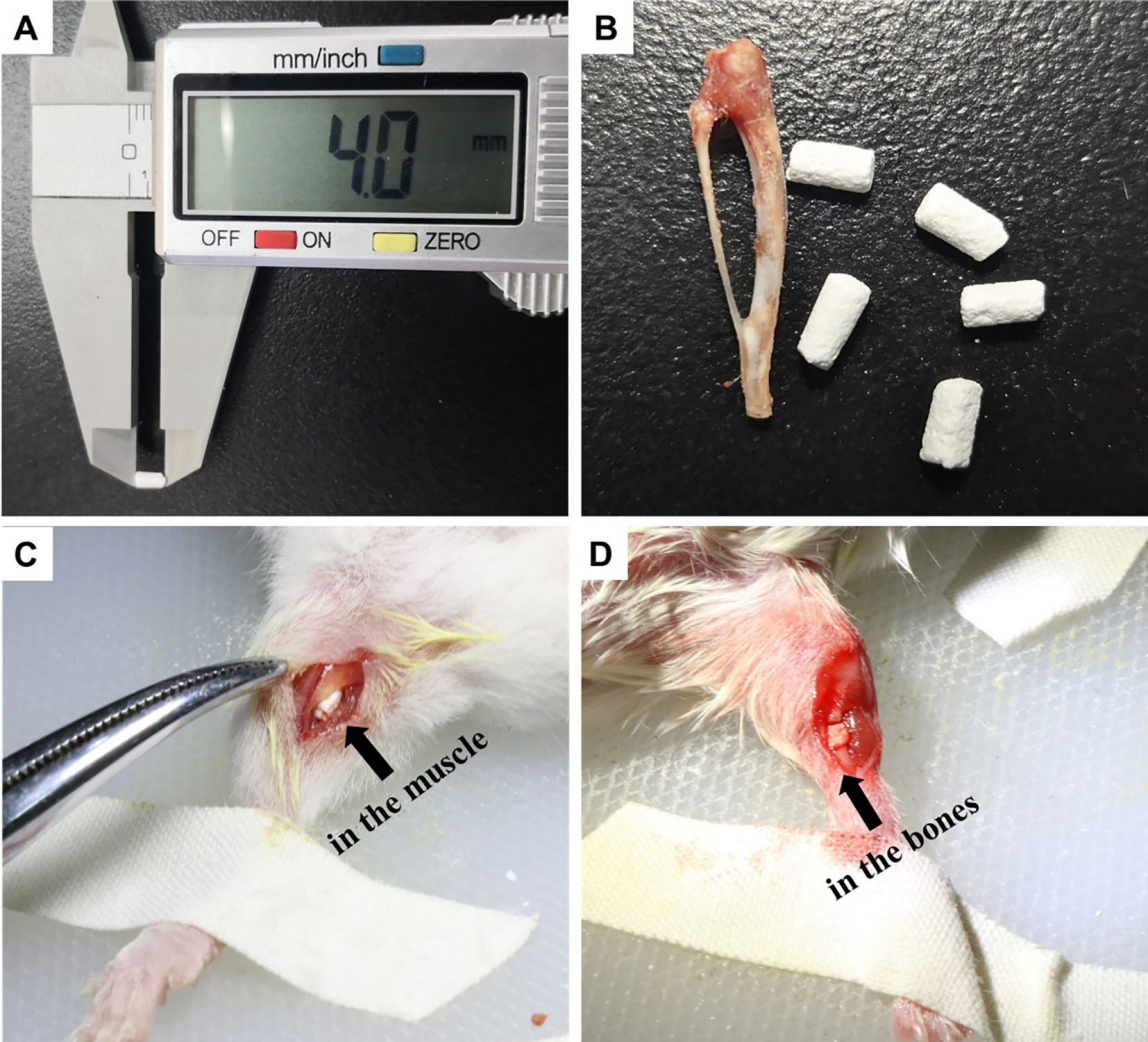
The material morphology and animal surgeries. (**A**) The length of the CTC composite used in our experiments was 4 mm. (**B**) The CTC and HA/TCP materials were shaped to resemble the tibia of mice. (**C**) The materials were implanted in the muscle of mice. (**D**) The materials were used to fill the large segment of the right tibia defect in mice.

Forty BALB/c mice were purchased from Dossy Biological Technology Company (Chengdu, China). All animals were maintained in a temperature and light-controlled environment ventilated with filtered air. In bone induction experiments, 20 mice were anesthetized with an intraperitoneal injection of pentobarbital sodium. The hair on both thighs was removed using an electric shaver and the skin underneath was disinfected with 75% ethanol, and then an approximately 10-mm muscle pouch was made which was parallel to the femur. Finally, a Ф2 $\times$ 4 mm CTC composite or HA/TCP was implanted into the muscle pouch of the left and right thighs of ten mice (n = 20), respectively (Fig. 3A–C). In the bone defect experiments, twenty mice were also anesthetized with hair removal and disinfection, and then the an approximately 10-mm longitudinal skin and muscle incision in the right leg was made. Next, a 4 mm diaphysis and periosteum were removed from the middle tibia to prepare the bone defect model, and the CTC or HA/TCP materials that were similar to the removed tibia were transplanted into the defect area in ten mice (n = 10), respectively (Fig. 3D). In the above two surgeries, the incised muscle and skin were closed with nylon sutures, and penicillin was injected intramuscularly to prevent infection lastly.

The study was carried out in compliance with the ARRIVE guidelines. The Animal Care and Use Committee of Chengdu University approved the study. The operative procedures and animal care were performed in compliance with NIH guidelines on the care and use of laboratory animals, under the supervision of a licensed veterinarian.

### Radiological evaluation

The mice of two experiments under anesthesia were performed the conventional X-rays with an X-ray machine (GE, USA) in the First People’s Hospital of Chengdu, China at week 6. The distance of the X-ray source to the mice was 50 cm. The setting of the machine was 25 kV, 100 mA, and 160 mAs. Each mouse was exposed in face up and back down directions. Two radiologists with no prior knowledge about the experiment estimated the X-ray results.

### Histological staining

All the animals survived until the end of the experiment period (8 weeks); the CTC and HA/TCP materials (n = 20) implanted in muscle were harvested, containing the material and the surrounding muscle at week 8; and the two types of materials filling bone defects (n = 10) were also harvested, containing the material and the surrounding bone (the entire tibia) since the material and bone were hard to be separated at week 8. All samples were fixed in 10% neutral formalin buffer solution for approximately 24 h at room temperature, decalcified in 10% ethylene diamine tetraacetic acid (EDTA, pH 7.0) for about 20 days at room temperature, dehydrated and embedded in paraffin. The embedded samples were cut into 5-μm thick histological sections. Then they were stained with hematoxylin–eosin (HE), sarranine-fast green (SFG), toluidine blue (TB) and methylene blue-basic fuchsin solution (MBFS) with the corresponding kits in accordance with the manufacturer’s instructions.

### Histomorphometry

The slides were observed by optical microscopy, and the slides with ectopic bone formation in bone induction experiments were scanned with a NanoZoomer Digital Pathology scanner (NDP, Japan). The areas of new bone growth and total tissues were manually measured with our own software; ultimately, the area percentage of new bone tissues was calculated as the ratio between the total of the new bone area and the total tissue area.

### Tartrate-resistant acid phosphatase staining

The sections of bone defect experiments were dewaxed to water and placed in tartrate-resistant acid phosphatase (TRAP) incubated buffer (1 ml naphthol AS-BI phosphoric acid solution, 0.1 ml fast garnet GBC base solution, 9 ml TRAP buffer) at 37 °C for 1 h; after the sections were washed, the nucleus was stained with hematoxylin for 3 min; lastly, the sections were dehydrated with a gradient of ethanol solutions, transparent, dried, and mounting.

### Micro-computer tomography (μ-CT) scanning

Three samples of the CTC and HA/β-TCP groups in bone defect experiments were randomly selected to perform μ-CT scanning. After fixation in neutral formalin buffer solution, the samples were stored in alcohol and sent to scan μ-CT by a high-resolution Skyscan 1174 (Bruker, Belgium) at 10-μm voxel resolution and 55 kV. For three-dimensional analysis by μ-CT, 400 images of each sample were three-dimensionally reconstructed with the best threshold, and the overall and cross-section images were produced with our own software. In the meantime, the overall osteogenesis and bone trabecula were automatically analyzed through the value of total volume (TV), bone volume (BV), BV/TV, structure model index (SMI), trabecular thickness (Tb.Th), trabecular number (Tb.N), trabecular separation (Tb.Sp) and bone mineral density (BMD).

### Statistical analysis

Data were expressed as means ± standard deviation and analyzed by paired ANOVA (SPSS 13.0, SPSS, USA). A *P* < 0.05 was considered statistically significant.

## Results

### The characteristics of the CTC composites

#### Micro morphology

**Figure 4:**
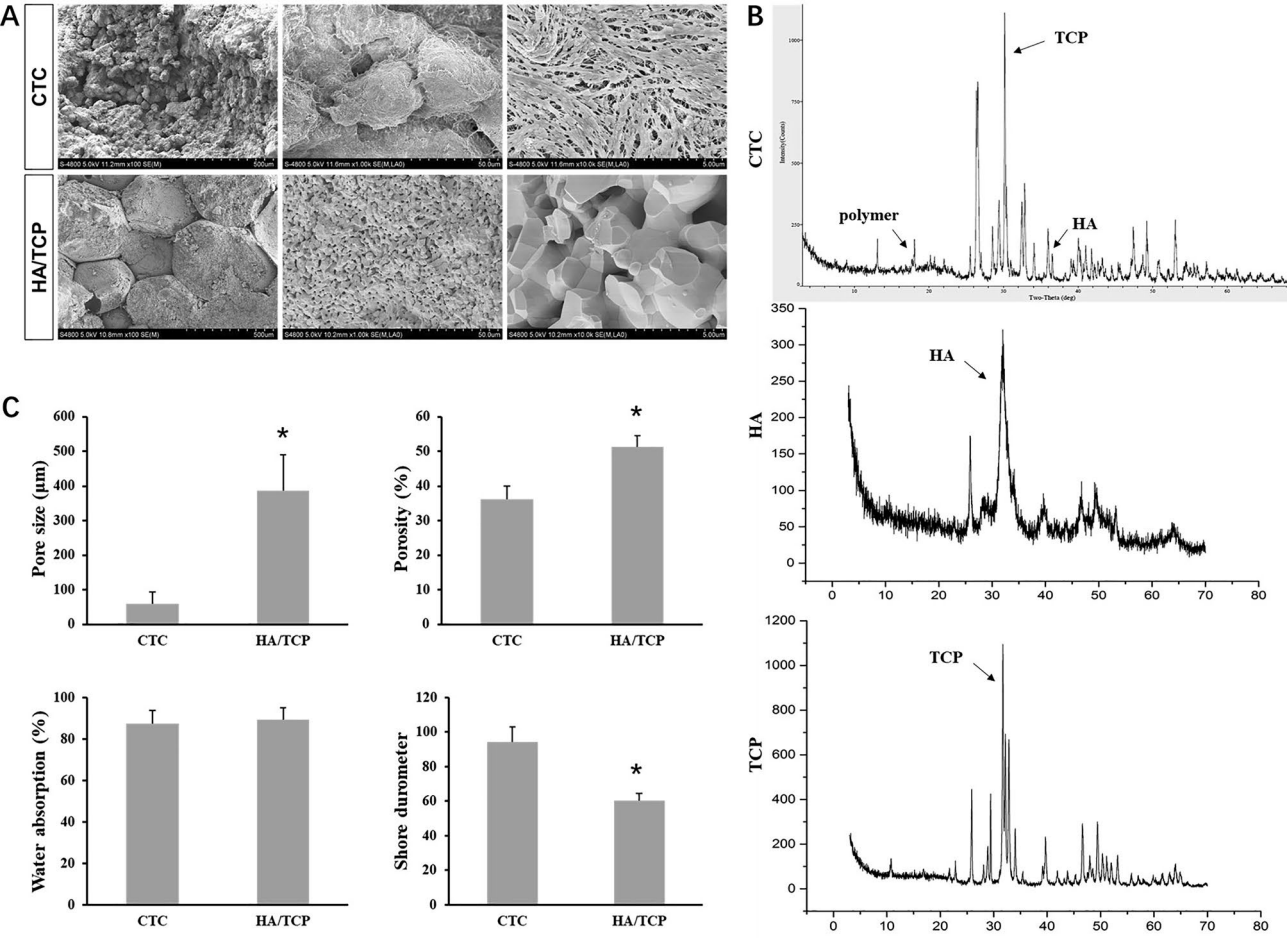
The physicochemical characteristics of the CTC and HA/TCP biomaterials. (**A**) The SEM pictures of the CTC and HA/TCP biomaterials with a magnification of 100, 1000 and 10,000 times. (**B**) The XRD spectrum of the CTC, HA and TCP materials indicated that the peaks represented polymer, HA and TCP, respectively. (**C**) Statistical data showed the pore size, porosity, water absorption and Shore durometer of the CTC and HA/TCP biomaterials, *P < 0.05.

Both the CTC and HA/TCP biomaterials were scanned by SEM. The SEM photos showed that collagen and hydrogel were mixed with CaP in CTC bio-composites, especially in the photo of higher proportions (10,000×), which presented the materials wrapped by hydrogel and formed many tiny pores (< 5 μm). On the other hand, there were also many pores in the compared HA/TCP biomaterials, but no proteins or hydrogel were observed in any magnification $(\times 100; 1000\,\text{ and} \,10,000)$, which are shown in Fig. 4A.

#### Chemical composition

The CTC composites were composed of PLGA-PEG-PLGA polymer, tricalcium phosphate and type I collagen. The X-ray diffraction (XRD) spectrum presented polymer and calcium phosphate, it is a pity that the collagen protein couldn’t be identified by XRD (Fig. 4B). The XRD of pure HA and TCP were used as controls.

#### Pore size, porosity, water absorption and shore durometer

Except the tiny pores, the pore size of the CTC composites ranged from 20 to 100 μm, and the pore size of the HA/TCP was 300–500 μm according to the SEM micro photos (Supplementary Figure S1); the volumetric porosity of the CTC composites was approximately 36.25% and that of HA/TCP was about 51.26% based on liquid displacement method (*P* < 0.05). The pore size and porosity affect the hardness and osteogenic properties of the biomaterials. There was no significant difference about the water absorption between the CTC and HA/TCP biomaterials (*P* > 0.05). The Shore hardness of the CTC and HA/TCP biomaterials were 94.35 and 60.38, respectively (*P* < 0.05), which indicated that the CTC bio-composites had higher hardness than HA/TCP biomaterials (Fig. 4C).

### Bone induction experiments

#### Observation of implanted materials with radiography in vivo

**Figure 5:**
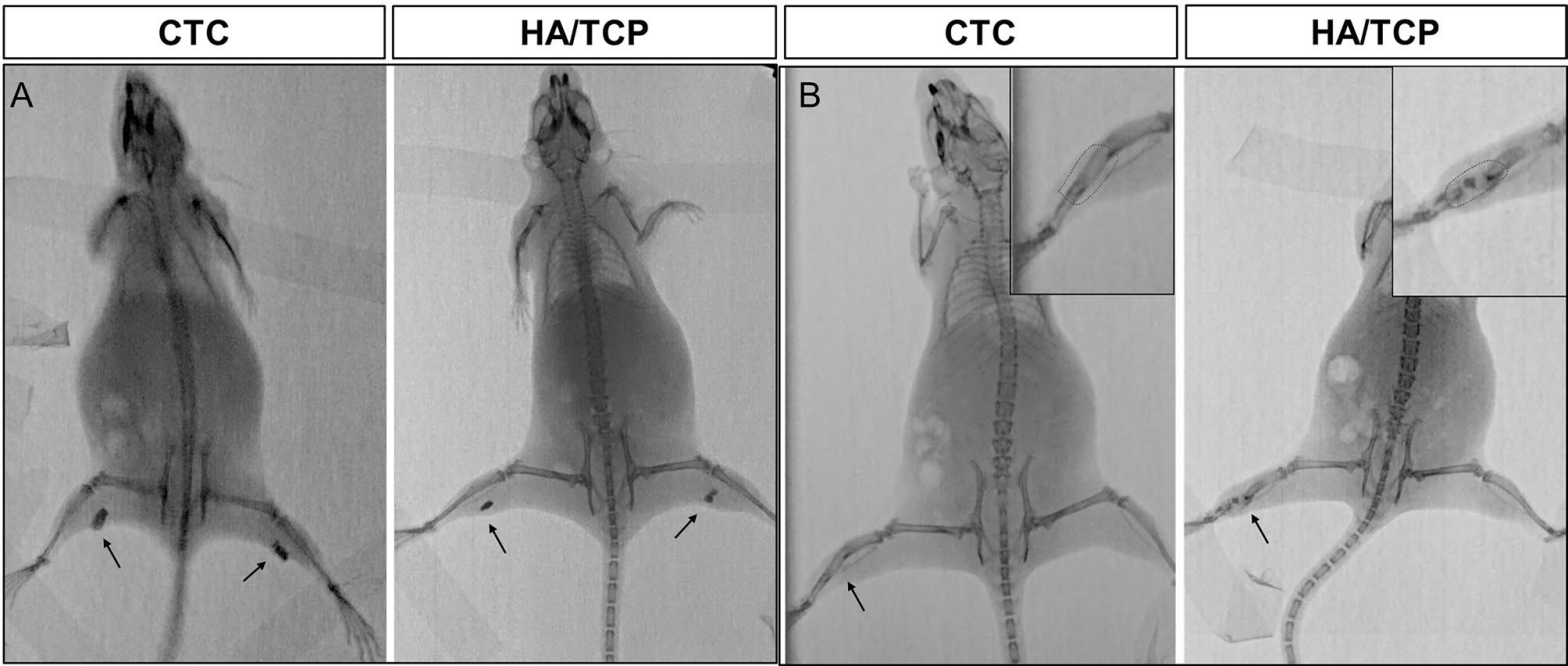
Detection of materials with radiography in vivo. (**A**) The X-ray photography of the CTC and HA/TCP biomaterials implanted in muscles of bilateral thighs in mice; arrow: the implanted materials. (**B**) The X-ray photography of the CTC and HA/TCP biomaterials filled in the right tibia defects in mice; arrow: bone defect sites; red dotted box: implanted material sites.

The frequently used HA/TCP biomaterials have good osteoinductivity which had been proved in our previous studies^16^. To verify the osteogenic ability of the CTC bio-composites, the composites were implanted into the muscle (non-osseous sites) of mice while the HA/TCP biomaterials were set as control. Six weeks after surgery, all mice in both of the two groups were scanned by X-ray, which showed a shadow in the muscle of the thigh indicating that the materials were well fixed in muscle and kept some distance from the femur or tibia (Fig. 5A). Unfortunately, there were no significant differences in shadows between the two types of materials. However, the materials showed low density shadow (dark grey), and the bone tissues of mice itself showed high density shadow (light grey).

#### The osteoinductive ability of implanted materials

**Figure 6:**
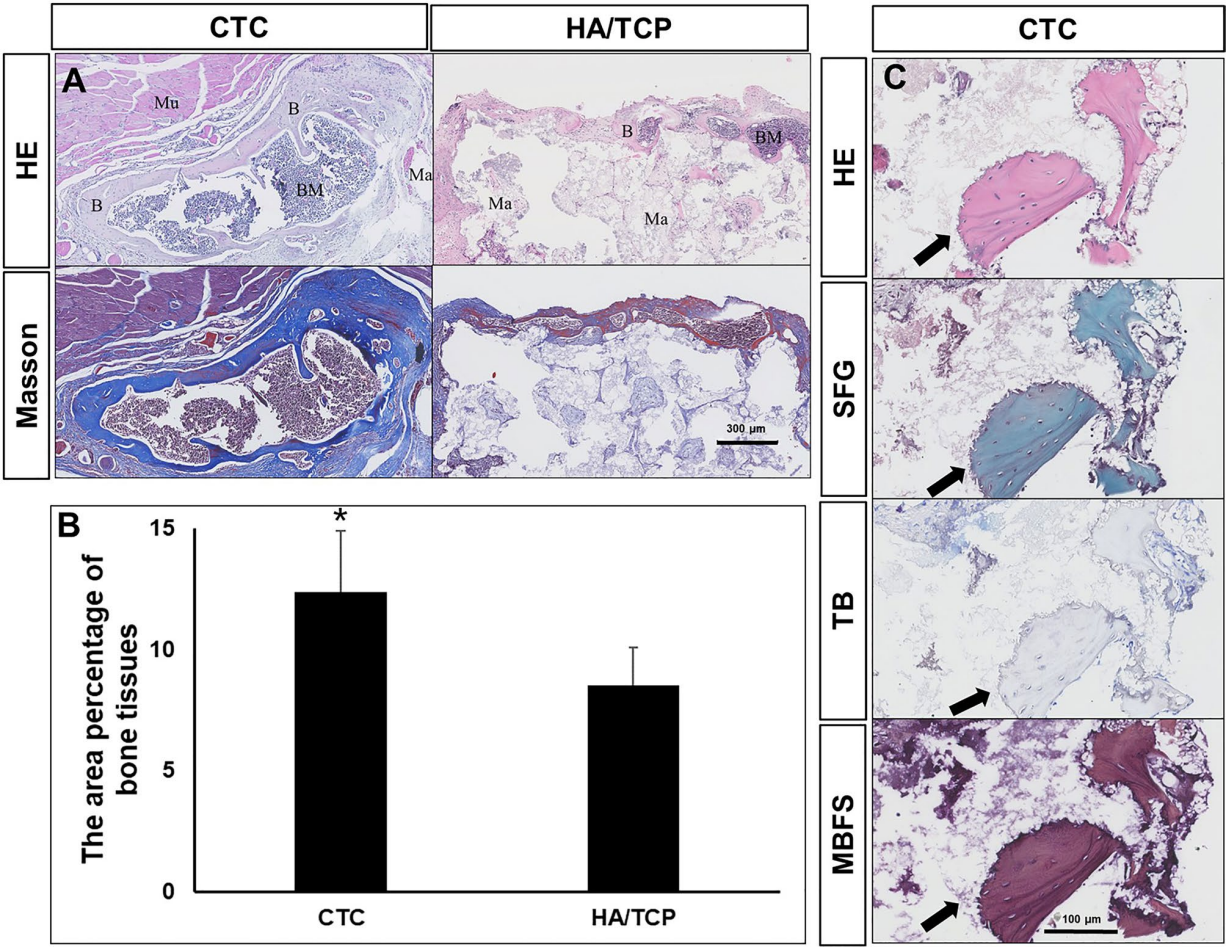
The microstructure of implanted materials and the area percentage of new bone tissues. (**A**) HE and Masson’s trichrome staining photos showed the microscopic details of samples. In HE photos, the bone tissues were stained pink, and bone marrow cells were dark blue. In Masson’s trichrome staining, the collagen fibers were stained blue, while the mature bone tissues were stained red. *B* bone tissues, *BM* bone marrow, *Mu* muscle, *Ma* materials, Bar: 300 μm. (**B**) The area percentage of new bone tissues was (12.38 ± 2.52) % in the CTC group, and (8.52 ± 1.57) % in the HA/TCP group, *P < 0.05. (**C**) The serial sections of hematoxylin–eosin (HE), sarranine-fast green (SFG), toluidine blue (TB) and methylene blue-basic fuchsin solution (MBFS) staining showing only bone tissue, no cartilage was observed during the osteoinduction. Arrow: bone tissues; bar: 100 μm.

To analyze the differences of bone induction between the CTC and HA/TCP biomaterials, the histological staining, HE and Masson’s trichrome, were performed to estimate the microscopic details 8 weeks after surgery. The results showed that the CTC bio-composites had a better osteoinductive ability, which could induce larger areas of bone and bone marrow tissues than that of HA/TCP materials. The surrounding muscle and the non-degraded materials were still observed (Fig. 6A). More enlarged pictures revealing osteoblasts and osteocytes were shown in Supplementary Fig. S2. The area percentage of new bone tissues were (12.38 $\pm$ 2.52)% in the CTC group, which was significantly higher than that in the HA/TCP group (8.52 $\pm$ 1.57)%, (*P* < 0.05, Fig. 6B). Where the area of bone tissues was the key factor in bone induction, the larger the areas of bone tissues, the stronger bone function.

#### Identification of the osteogenic way with three special staining

To identify the way of osteogenesis, the special staining of SFG, TB and MBFS was performed to identify the bone tissues or cartilaginous tissues, which was intramembranous ossification or endochondral ossification. The cartilage is stained red and the bone is stained green in SFG staining. Our results only showed green bone tissues without cartilage tissues; there was also no purple cartilage in the field of vision in TB staining; and the osteoblasts were stained dark blue and the bone was stained red in MBFS staining (Fig. 6C). All the three staining methods showed no cartilage formation, indicating that the osteogenic way of osteoinduction was through intramembranous ossification, not endochondral osteogenesis.

### Bone defect experiments

#### The repairing effects of bone defects with radiography in vivo

To detect the repairing effects of 4 mm-tibia defect in live mice, all mice were taken X-ray photographs under anesthesia 6 weeks after surgery. From the bone shadow photography based on X-ray, the material and bone were completely integrated into a continuous tibia since lots of new bone tissues were induced by the CTC composites, and no fracture line was found. On the contrary, the bone defects were not fully healed with the HA/TCP biomaterials, since there was no continuous tibia, and some messy low-density shadow was formed at the defect site, the HA/TCP biomaterial was extruded by the growth of tibia and broke into clumps within the bone defect site (Fig. 5B). The negative control (without filling materials) showed an unhealed defect by X-ray in another supplementary experiment (Supplementary Fig. S3). The results indicated that the CTC composites had better repairing effects than the HA/TCP biomaterials.

#### Detection of repairing effects by histological staining

**Figure 7:**
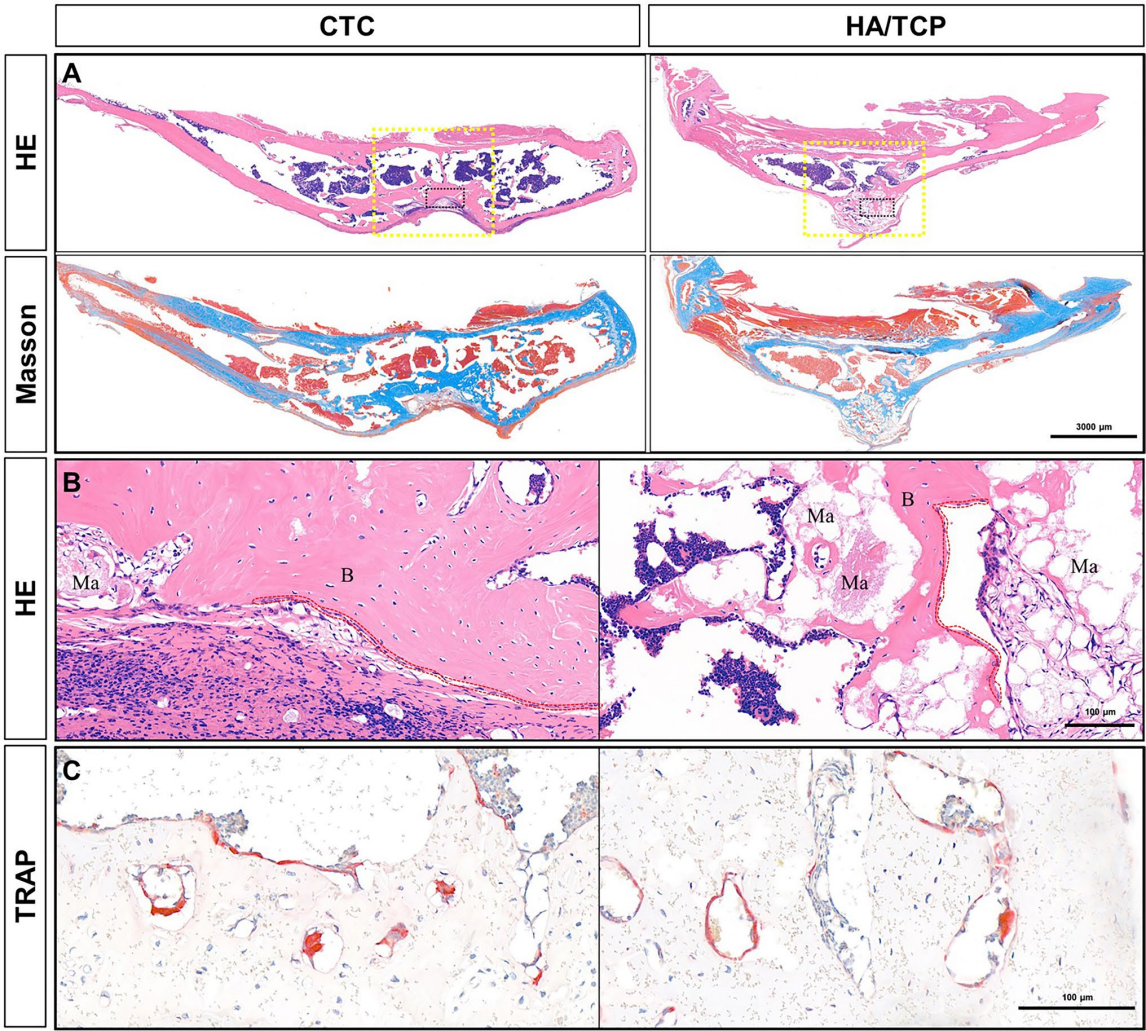
The microstructure of repairing tibia defects in mice. (**A**) The HE and Masson’s trichrome staining showing the bone repair after filling the 4-mm tibia defects with the CTC composites and HA/TCP biomaterials. Yellow dotted box: the implanted material sites; black dotted box: being enlarged to figure B; bar: 3000 μm. (**B**) Enlargement of figure A, observation of osteoblasts bone tissues and materials, red dotted line: osteoblasts; *B* bone tissues, *Ma* non-degraded materials; bar: 100 μm. (**C**) The TRAP staining showed osteoclast formation in the new bone tissues, bar: 100 μm.

The entire tibia of mice containing materials was stripped off, then decalcified, embedded, and serially sliced. Serial sections were performed by HE and Masson's trichrome staining. In the CTC group, the bone defects were well repaired, and the newly formed bone and the original bone were very well integrated; since the bio-composites were degraded gradually and replaced by new bone tissues 8 weeks after surgery, it would only take time to recover. In the control group, the biomaterials were extruded by the new bone of self-healing not induced by materials as their low hardness of HA/TCP biomaterials. There were still many non-degraded materials being dyed pink (Fig. 7A). The results indicated that the CTC composites had better bone repairing and load-bearing capacities than the HA/TCP biomaterials determined by histological observation.

#### The balance of osteoblasts and osteoclasts

By enlarging the image of bone healing on the HE sections, the osteoblasts were observed linearly arranged along the newly formed bone tissues (Fig. 7B). To further clarify the relationship of bone formation and resorption, we also detected the osteoclasts in the newly formed bone tissues. Using the same samples as before, the osteoclasts were detected in the mature bone tissues that were stained red with TRAP staining (Fig. 7C); it seemed to form the bone marrow tissues to render certain the balance of bone reconstruction and resorption.

#### Reconstruction of bone defect repairing with micro-CT

**Figure 8:**
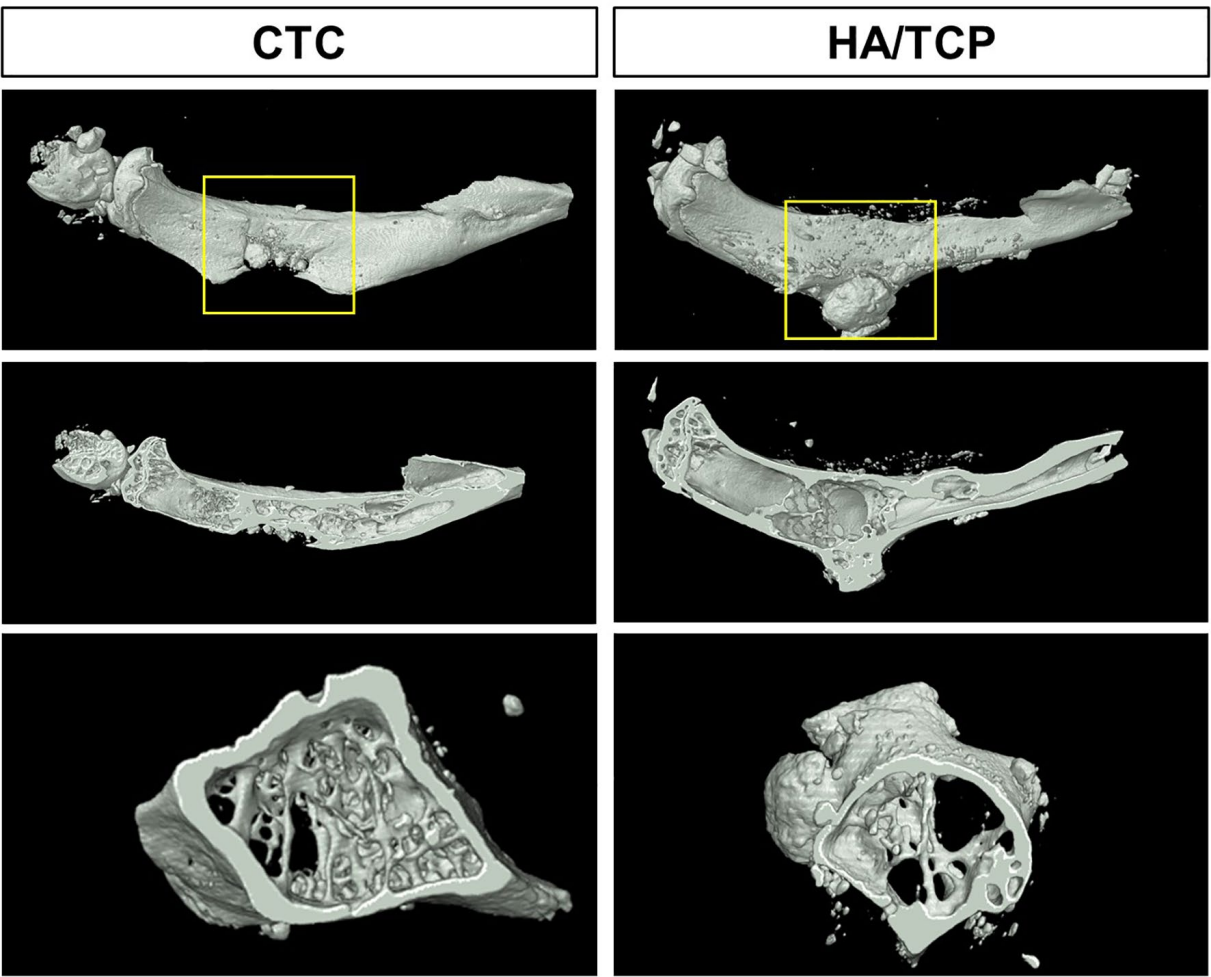
Three-dimensional reconstruction of bone defect repairing by the CTC and HA/TCP biomaterials at week 8 by micro-CT. The yellow boxes were chosen as a region of interest (ROI) to analyze the data of osteogenesis and trabecula.

To reconstruct the real scene of bone repair in vivo, the micro-CT was used to reveal the specific and visualized repair results 8 weeks after surgery. The stereogram, profile and transverse were captured to show the details. From these pictures, we could see that the repair effects were significantly better in the CTC group than that in the HA/TCP group (Fig. 8). The yellow boxes of Fig. 8 (implanted materials) were chosen as a region of interest (ROI) to analyze the data of osteogenesis and trabecula with built-in software of micro-CT (Table 1), for further analysis of bone repairing effects. The unoperated lateral tibia was set as positive control group (Supplementary Fig. S4). The results showed the CTC composites had better bone repairing effects than the HA/TCP biomaterials determined by parameters of osteogenesis.

**Table 1:** The parameters of osteogenesis in bone repair.

	TV (mm^3^)	BV (mm^3^)	BV/TV (%)	SMI	Tb.Th (mm)	Tb.N (1/mm)	Tb.Sp (mm)	BMD (g/cm^3^)
CTC	19.7308	10.2012	0.5175*	1.8477*	0.1609	2.0223*	0.6019	1.0675*
HA/TCP	24.1517	8.7092	0.3623	2.6660	0.1243	1.1847	0.8051	0.8634
Control	20.3096	14.0417	0.6928	1.5088	0.1766	2.9712	0.5414	1.6059

The control refers to the unoperated lateral tibia.

*TV* tissue volume, *BV* bone volume, *BV/TV* bone volume fraction, *SMI* structure model index, *Tb.Th* trabecular thickness, *Tb.N* trabecular number, *Tb.Sp* trabecular number, *BMD* bone mineral density.

The comparison between CTC and HA/TCP, *P < 0.05.

## Discussion

It is an acknowledgement that calcium phosphate bio-ceramics were excellent bone graft substitutes, especially in the cancellous bone of non-load bearing bones^17,18^; and the HA/TCP was a kind of typical CaP biomaterials which had good osteoinductivity^19^. However, it was proved that HA/TCP was not suitable for repair of large segments of load-bearing bone in this study. The composites based on CaP have always been the trend of development of artificial bone. Many researches were working in this direction including the addition of titanium^20^, PLA^21^, polycaprolactone (PCL)^22^, BMP^23^, collagen^24^, chitosan^25^, hyaluronic acid^26^, and/or alginate^27^, and other similar materials. To enhance the mechanical property, the polymer was the preferred choice since it could be degraded in vivo, and its degradation product could be converted into pyruvate, which entered the three-carbon cycle and was eventually expelled as CO_2_^28,29^. Therefore, the polymer could be used as the components of CaP based composites to improve the elasticity modulus. The thermosensitive hydrogel (PLGA-PEG-PLGA polymer) used in this study was a kind of high-performance polymers that could be the binding material except for the degradable materials. To improve the osteogenic ability of composites, collagen was the top-priority choice since it was the main protein secreted by osteoblasts, which also have been proved excellent osteogenic property^30,31^. Our study used the type I collagen from rat tail to synthesize the collagen-thermosensitive hydrogel-calcium phosphate bio-composites, which could improve osteogenesis as well as reduction of immunological rejection in mice. In the future work, we will replace rat collagen I with recombinant human collagen I. Glutaraldehyde is a commonly used crosslinking agent for porous scaffolds, it is toxic, but we use low doses of 2.5% glutaraldehyde, and it has been dissolved and washed away by 95% alcohol in our study. Our results revealed that CTC bio-composites had better mechanical property and less porosity than that of the HA/TCP biomaterials, indicating that the CTC composite was more suitable for filling load-bearing bones. Further, we also proved that the osteogenic ability of CTC composites was stronger compared to the conventional CaP biomaterials.

Induction of ectopic bone formation in the non-osseous sites, such as muscle, by the CaP biomaterials, was defined as osteoinduction^19^. It has been proved that the chemical composition (Ca or P) was the prerequisite in osteoinduction^32^, which was consistent with this study. The CTC bio-composites containing CaP have induced ectopic bone formation. It remains difficult to repair the large segments of bone defects in orthopedics owing to tumor resection, serious bone trauma, etc*.* The size of critical-sized bone defects has been considered as the focus of controversy^33,34^, whether the defect healed by its own growth also depended on the host; therefore, we chose a 4-mm tibia defect and filled with the CTC composites or HA/TCP biomaterials in this study, and the repair efficacy was better with CTC composites. On the other hand, it is worth noting: the materials were not fixed with bone screws after implantation, since the tibia of mice were too small to be fixed; however, the material was not shifted as setting close to the muscle. This measure not only makes the operation simple, but also achieved a good repair effect.

## Conclusions

According to the above methods and results, our study proved that the CTC bio-composite had better mechanical property, osteoinductive and bone repairing ability than the conventional HA/TCP biomaterials, indicating the CTC composites exert good weight bearing capacity during repairing large segments of tibia defects in mice; while the HA/TCP biomaterial was not suitable for weight bearing bone defects. This study provides a new thought for the synthesis of artificial bone from the perspective of raw materials, and an ideal option for future orthopedic patients.

## Supplementary Information

Supplementary Information.
