# Supplementary material for: The preparation and application of calcium phosphate biomedical composites in filling of weight-bearing bone defects
Source: Sci Rep. 2021 Feb 19;11:4283. doi: 10.1038/s41598-021-83941-3 (PMC7896074; doi:10.1038/s41598-021-83941-3)
Supplement: Supplementary file 1 — Supplementary Information. [file 41598_2021_83941_MOESM1_ESM.pdf]

The Preparation and Application of Calcium Phosphate Biomedical Composites in Filling of Weight-Bearing Bone Defects

Lijia Cheng[1,*], Tianchang Lin[1], Ahmad Taha Khalaf[1], Hongyan He[1], Liming Yang[2], Shuo Yan[1], Jiang Zhu[1], Zheng Shi[1,*]

[1]College of Basic Medicine & Affiliated Hospital/Clinical College, Chengdu University, Chengdu, 610106, China

[2]Department of Orthopedics, the First People's Hospital of Chengdu, Chengdu, 610000, China

*Co-corresponding authors: Lijia Cheng (chenglijia@cdu.edu.cn) and Zheng Shi (drshiz1002@hotmail.com)

Supplementary figures:

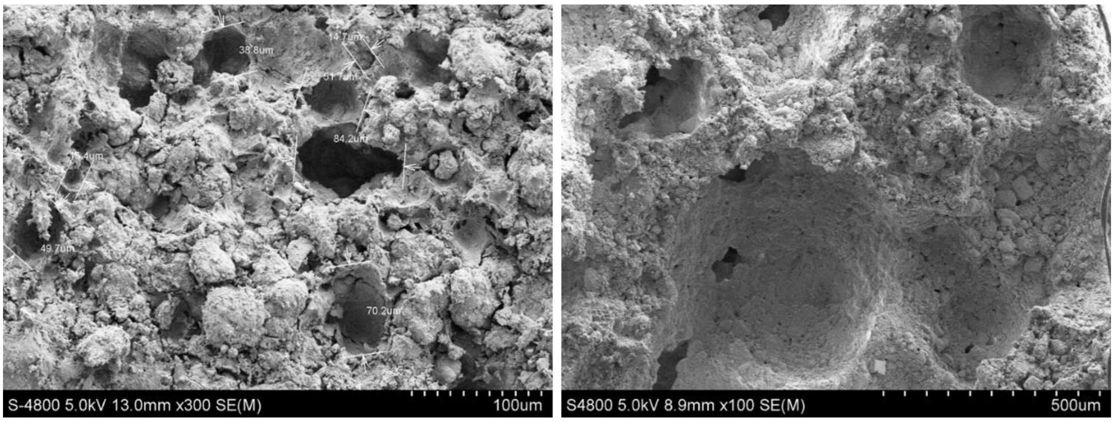

Supplementary Figure S1: The SEM micro photos showed the pore size of the CTC composites (left) and HA/TCP biomaterials (right).

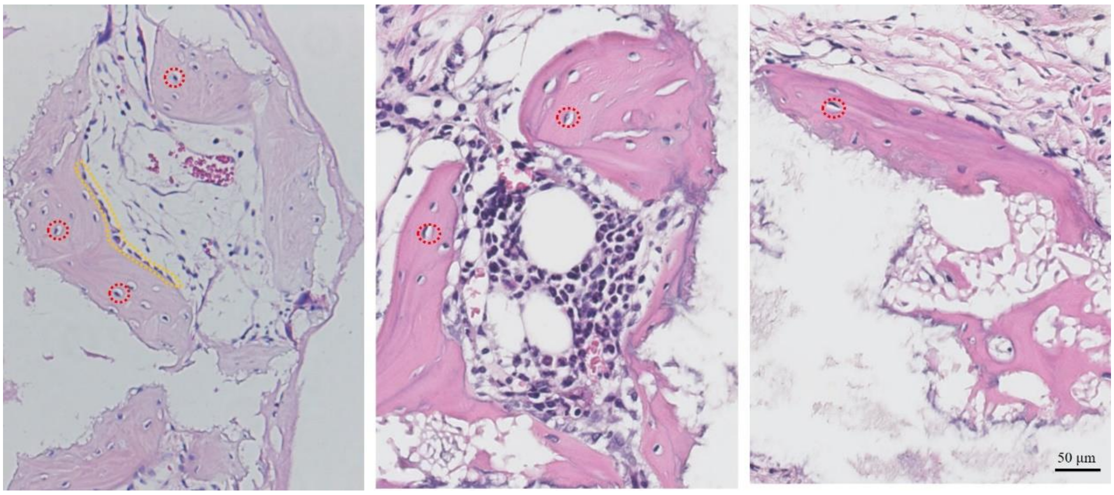

Supplementary Figure S2: The HA/TCP biomaterials were implanted in muscle of mice and harvested at 8 weeks, HE staining showed new bone tissues, osteoblasts and osteocytes in the materials, which proved the osteoinduction occurrence in mice. Red dotted box: osteocytes; orange dotted box: osteoblasts.

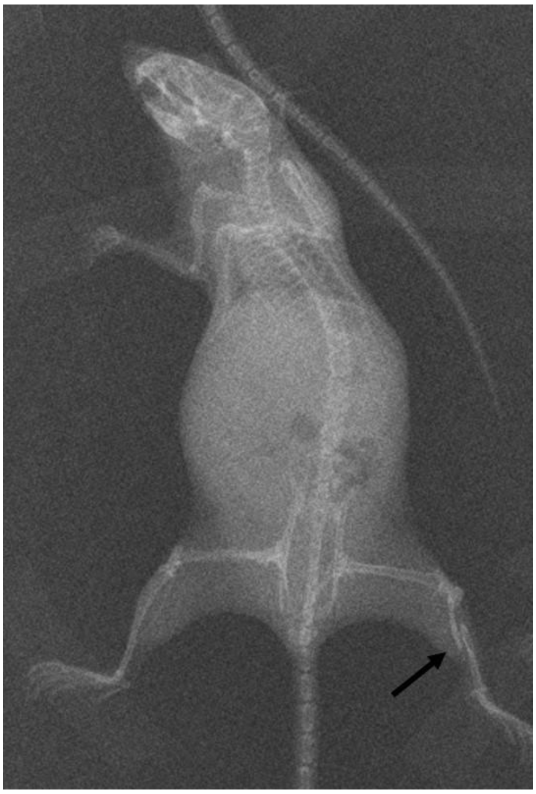

Supplementary Figure S3: A 4 mm defect was prepared and no material filling in the defect (the negative control group) in a supplementary experiment. Six weeks later, the X-ray showed an unhealed defect in the operative site.

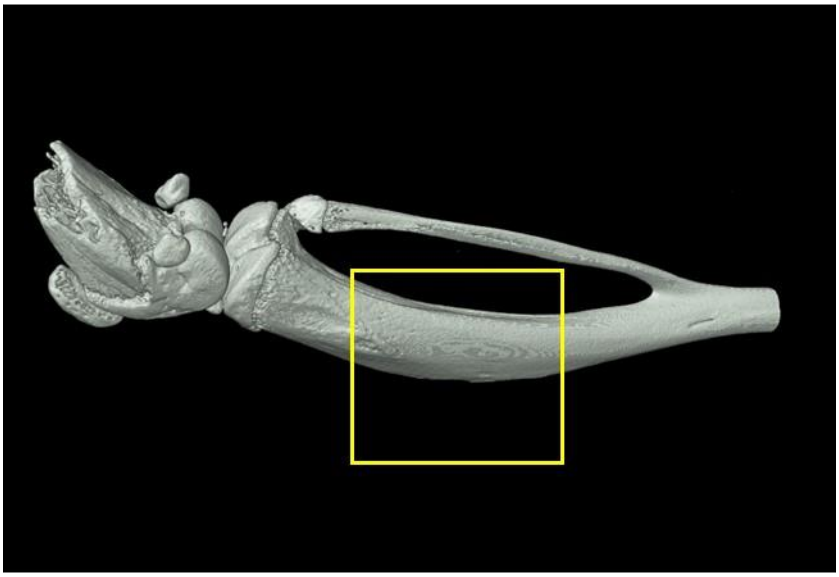

Supplementary Figure S4: The unoperated tibia of mice (the positive control) was three-dimensionally reconstructed by micro-CT, the yellow box was chosen as a region of interest to analyze the data of TV, BV, BV/TV, SMI, Tb.Th, Tb.N, Tb.Sp and BMD with built-in software of micro-CT.
